# Techno-strain and techno-insecurity are associated with poor mental well-being in specific age and occupation groups

**DOI:** 10.1093/joccuh/uiae079

**Published:** 2024-12-27

**Authors:** Hang-Ju Yang, Yawen Cheng, Yen-Ling Liu, Wan-Ju Cheng

**Affiliations:** Department of Emergency Medicine, Jen Ai Hospital Dali Branch, 483 Dongrong Rd, Dali, Taichung, Taiwan; College of Public Health, Institute of Health Policy and Management, National Taiwan University, 17 Xuzhou Rd, Taipei, Taiwan; National Center for Geriatrics and Welfare Research, National Health Research Institutes, 35 Keyan Rd, Zhunan, Miaoli County, Taiwan; National Center for Geriatrics and Welfare Research, National Health Research Institutes, 35 Keyan Rd, Zhunan, Miaoli County, Taiwan; Department of Psychiatry, China Medical University Hospital, 2 Yude Rd, Taichung, Taiwan; Department of Public Health, China Medical University, 100 Sec. 1, Jingmao Rd, Taichung, Taiwan

**Keywords:** burnout, depression, information and communication technology, occupational health, work conditions

## Abstract

**Objectives:**

Innovative technology at work can lead to stress and has been linked with adverse work and health consequences. This study aimed to examine the association of techno-insecurity and techno-strain with mental well-being in different age and occupational groups.

**Methods:**

We used a nationally representative survey of the working population and restricted our analyses to 2814 employees who reported being engaged with new technology. Techno-insecurity and techno-strain were evaluated by a single question each. Mental health status was assessed by a 5-item scale, and burnout status was assessed by the Copenhagen Burnout Inventory. We used logistic regression analysis to examine the association of techno-insecurity and techno-strain with mental well-being, adjusting for job control, psychological demands, job insecurity, and workplace violence. We further stratified study participants by age and occupational group and examined the association in each group.

**Results:**

One-fifth of the study participants reported having techno-insecurity and techno-strain. Techno-insecurity was associated with a 1.8-fold increased risk of poor mental health and high burnout, whereas techno-strain was associated with a 2.2-fold increased risk of having poor mental health and high burnout. The associations between techno-insecurity or techno-strain and poor mental health were most profound among middle-aged workers. Among all occupational groups, the associations between techno-insecurity or techno-strain and burnout were most profound among manual workers.

**Conclusions:**

Techno-strain and techno-insecurity are emerging occupational mental health threats, particularly among middle-aged and manual workers. To promote mental health, resources provided by the organization are needed to help employees cope and work with technology.

## Teaser

Key points

What is already known on this topic:

• Technology-related stress is an emerging occupational health concern, with previous studies linking it to decreased productivity and psychological symptoms in certain occupations.

What this study adds:

• In a nationally representative survey of employees across various occupations, techno-insecurity and techno-strain were associated with poor mental health and burnout.

• These health effects were most pronounced in middle-aged and manual workers.

How this study might affect research, practice, or policy:

• Organizational resources are needed to support employees in coping with and working effectively alongside technology.

## 1. Introduction

With new technology’s increasingly pervasive use in the workplace, how technology-related stress affects workers’ health has become an emerging occupational health concern. Techno-stress is stress experienced by workers in organizations as a result of their use of technology,^1^ with ineffective coping leading to distress.^2^

### 1.1. Techno-stress

Five components have been proposed to conceptualize techno-stress: techno-invasion (constant connectivity that invades aspects of life beyond work), techno-overload (increased pace and volume of work due to technology), techno-complexity (technology that users find difficult to understand), techno-insecurity (fear of job loss to technology or to others with greater technological competency), and techno-uncertainty (ambiguity around expectations related to technological changes); each have their own effects on health and work outcomes.^3^ In business literature, techno-stress is associated with lower productivity and decreased job satisfaction.^4^ In regard to occupational health and safety, techno-stress has been observed to be related to psychological symptoms, including fatigue, insomnia, and irritability.^5^ Nevertheless, studies on the association between techno-stress and workers’ health have been limited by recruiting participants using convenience sampling and from single occupations or industry, and by restricting participants to highly educated workers.^6^

### 1.2. Techno-strain

Techno-strain is the degree to which an employee feels strained due to technology usage in connection with work tasks.^7^ It encompasses anxiety, fatigue, skepticism, and feelings of inefficacy related to technology use.^8^ Moore^9^ focused on the aspect of fatigue of techno-strain and constructed 4 items for its measurement: feeling drained from activities that require the use of technology, feeling tired from technology-related activities, perceiving that working all day with technology is a strain, and feeling burned out from technology tasks. Techno-strain has been observed to associate with intentions to leave one’s job among technology professionals.^9^ On the other hand, the Job Demand-Control (JD-C) model, proposed by Karasek and Theorell,^10^ has been widely used to assess psychosocial job strain; this model defines high job strain as a combination of high job demands and low job control, which has been observed to lead to burnout^11^ and poor mental health.^12^ However, how techno-strain differs from general psychosocial job strain and how it relates to burnout or poor mental health have not been examined.

### 1.3. Aging and technology at work

Demographic changes in developed countries have led to the aging of working populations.^13^ A report by the World Bank^14^ highlights that the East Asian and Pacific region is aging faster than other global regions. In Taiwan, for example, the percentage of people aged over 65 was above 14% in 2018 and is expected to rise above 20% by 2025. Older adults find it more difficult to handle technologies than younger people due to a lower perceived ease of use.^15^ Among office workers, age also increases negative perceptions of technological changes, as there are fears that computerization may pose threats to their own employment, along with a lack of clear knowledge of how computers operate.^16^ Nevertheless, a review study concluded that the use of information and communication technology was associated with burnout in middle-aged employees but not in older employees.^17^ This may be because the family structure of middle-aged workers can complicate the work–life balance, whereas older workers have life experiences that help them cope with techno-stress. Furthermore, among the 5 components of techno-stress, techno-insecurity may impact workers differently by age. Older workers, who are less likely to be re-employed after layoffs and more prone to long-term unemployment, may experience a greater mental impact from techno-insecurity compared with younger workers.^18^ Empirical studies are needed to explore how employees of different ages cope with technology and how their mental well-being is affected by it.

### 1.4. Health impact of technology in different occupations

There is a substantial body of literature on the impact of job automation on employees across various occupations. Similarly, the extent to which new technology affects different occupations can vary. For instance, the growing use of service and collaborative robots has raised concerns about whether cognitive interactions between humans and robots might induce stress and pose risks to workers’ mental and physical health.^19^ Research indicates that employees in jobs with a higher likelihood of automation report greater job insecurity, which is in turn linked to poorer health outcomes.^20^ Additionally, new technology has been observed to be associated with an increased risk of losing paid employment among older employees working with people but not among those working in the offices or in production roles,^21^ suggesting that the impact varies by occupation. However, previous studies examining different occupations have not directly assessed insecurity specifically related to the introduction of new technology at work, known as techno-insecurity.

### 1.5. Study aims

**Figure 1:**
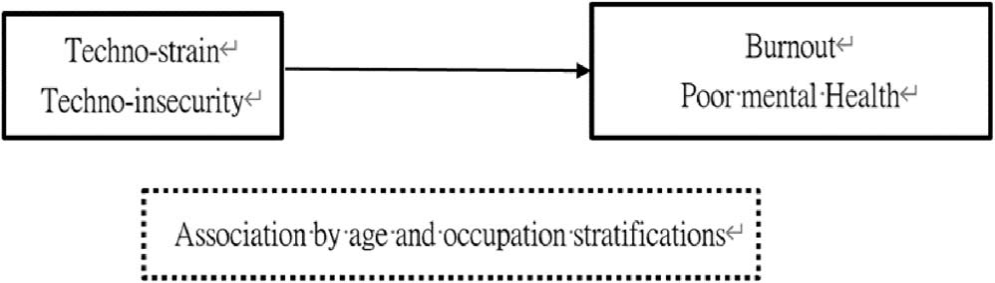
Study framework.

To date, studies on the association of techno-strain and techno-stress with workers’ mental health have been scarce, and these studies have been limited by a narrow range of occupations and small sample sizes.^3^ In this study, we used data from a national survey representing employees across a wide range of age and occupations. Our aim was to examine how the association of techno-strain and techno-insecurity—1 of the 5 components of techno-stress—with burnout and poor mental health among workers varies by age and occupation (Figure 1). We hypothesized that the association is positive and is more profound in older workers and low-skilled workers.

## 2. Methods

### 2.1. Participants

The Ministry of Labor of Taiwan has conducted nationwide surveys of the working population every 3 to 5 years since 1988 to understand safety and health conditions in the workplace. In this study, we used the 2022 survey, which included native workers who participated in labor insurance. To generate a representative sample, systematic sampling was conducted by stratifying workers by living area, occupation, and industry. The survey was completed in Mandarin for 4009 participants by face-to-face interview, with a response rate of 76.6%. All participants provided informed consent. Individuals younger than 20 years (*n* = 23), employers (*n* = 181), the self-employed (*n* = 430), and unpaid family workers (*n* = 33) were excluded. To examine the impact of technology, we further excluded those who did not use digital devices at work (including desktop computers, the internet, mobile devices, automated machinery and equipment, robots, and communication software) (*n* = 528). Ultimately, a total of 2814 participants were included in the following analyses.

This study was approved by the ethics committee of National Taiwan University (202205HS092). Informed consent was waived because the secondary data did not contain any identifiable personal information.

### 2.2. Techno-insecurity and techno-strain

We measured techno-insecurity with 1 item: “I feel that new technologies threaten my job security.” The question was adapted from a 3-item scale,^22^^,^^23^ with the other 2 items being “Technology will advance to an extent where my present job can be performed by a less-skilled individual” and “Technology makes it easier for other people to perform my work activities.” Techno-strain was assessed using a single item: “The use of new technology makes me feel tired.” This item was adapted from a 4-item scale,^9^ which also included the following items: feeling drained from activities that require the use of technology, feeling tired from technology-related activities, perceiving that working all day with technology is a strain, and feeling burned out from technology tasks. We selected single items for these measures due to the space limitations of the national survey questionnaire. For the 2 questions, participants chose from a 4-point Likert scale (“strongly disagree,” “disagree,” “agree,” or “strongly agree”). The responses “agree” and “strongly agree” were categorized into the techno-insecurity and techno-strain groups. The responses “strongly disagree” and “disagree” were categorized into the no techno-insecurity and no techno-strain groups.

### 2.3. Burnout and general mental health

General mental health was evaluated using the Brief Symptom Rating Scale (BSRS-5), which assesses mood symptoms over the past week.^24^ It consists of 5 items: (1) feeling tense or keyed-up, (2) feeling depressed or in a low mood, (3) feeling easily annoyed or irritated, (4) feeling inferior to others, and (5) having trouble falling asleep in the past week. Participants rated each item on a scale of 0 (not at all), 1 (a little bit), 2 (moderately), 3 (quite a bit), and 4 (extremely). A total score of 6 or higher was considered indicative of poor mental health.^25^ Burnout was assessed using the 5-item scale for personal burnout from the Copenhagen Burnout Inventory.^26^ Each item was rated on a 5-point Likert scale (1 = never to 5 = always). The summed scores were standardized to 0-100. The score was dichotomized, with a score of ≥50 indicating burnout,^27^ distinguishing these individuals from those with lower levels of burnout.

### 2.4. Psychosocial work conditions and other variables

Psychosocial work conditions have been shown to influence workers’ health,^28^ regardless of technology use at work. In examining the association of techno-insecurity and techno-strain with mental well-being, it is important to control for psychosocial work conditions, including job control, psychological job demands, shift work, job insecurity, and workplace violence. Job control and psychological job demands were assessed using the validated Mandarin version of the Job Content Questionnaire, which is based on the JD-C model.^29^ A 5-item questionnaire for the demands scale (work is fast, work is hectic, work is hard, must concentrate on the job for a long time, workplace is understaffed) and a 7-item questionnaire for the control scale (learning new things, nonrepetitive work, creative work, various tasks, can develop one’s abilities, allowed to make own decisions, opinion is influential) were used. Responses were recorded on a 4-point Likert scale (1 = strongly disagree to 4 = strongly agree), and a summarized score for each scale was standardized. We classified job control and psychological job demands into high and low groups based on their medians (55.56 and 53.33, respectively). The original Mandarin version of the job insecurity scale consists of 6 items.^30^ Due to survey length restrictions, only 1 item (“My job is secure”) was used, as in previous studies,^31^ and it was rated on a 4-point Likert scale (“strongly disagree,” “disagree,” “agree,” “strongly agree”). The responses “agree” and “strongly agree” were categorized into the no job insecurity group, whereas “strongly disagree” and “disagree” were categorized into the job insecurity group.

Participants provided information about their work schedule, indicating whether they had a regular shift (fixed day shift, afternoon shift, or evening shift), a rotating shift, or an irregular shift. Participants were grouped into 2 categories: non–shift work (fixed day shifts) and shift work (encompassing afternoon shifts, evening shifts, rotating shifts, and irregular shifts). Furthermore, participants were asked if they had experienced any of the following types of workplace violence within the 12 months prior to the survey: physical, verbal, psychological, or sexual harassment. Experience of any type of workplace violence was coded as having workplace violence.

Participants self-reported their sex, age, educational level, and occupation titles. Age was divided into 4 categories: 20-34 years old, 35-44 years old, 45-54 years old, and 55 years old and above. Educational level was categorized into 2 groups: high school and below, and college or above. Occupations were coded according to the 6th edition of the Standard Occupational Classification by the Ministry of Labor of Taiwan and were subsequently grouped into 6 categories: (1) managers, (2) professionals, (3) skilled nonmanual (eg, science, medical, and engineering support professionals), (4) low-skilled nonmanual (eg, administrative assistants, service, and sales staff), (5) skilled manual (eg, farmers, fishers, foresters, construction workers), and (6) low-skilled manual (eg, machine operators, assemblers, cleaners, packers).

### 2.5. Statistical analysis

We examined demographics, work characteristics, and mental well-being of participants within 4 age groups: 20-34 years, 35-44 years, 45-54 years, and 55 years and above. Differences were tested using chi-square tests for categorical variables and Mann-Whitney tests for continuous variables. Logistic regression analysis was employed to estimate the odds ratio (OR) and 95% CI of techno-insecurity and techno-strain on poor mental health and burnout. The model was adjusted for sex, age, education level, occupation categories, shift work, psychological job demands, job control, job insecurity, and workplace violence. Further stratified analysis was conducted to examine the results of each logistic regression analysis across the 4 age groups and 6 occupational groups, and the results are shown using forest plots. Data analyses were performed using SAS 9.4 (SAS Institute, Cary, NC, USA).

## 3. Results

Of 2814 participants, 565 (20.08%) reported techno-insecurity and 573 (20.36%) reported techno-strain. A lower percentage of older study participants reported burnout than younger workers (9.2%, Table 1). Workers aged 45-54 more frequently reported techno-strain than workers younger than 35 years (23.5% vs 15.4%). The percentages of poor mental health and techno-insecurity were not significantly different between age groups.

**Table 1:** Demographic and work characteristics of study participants.

**Characteristic** ^**a**^	**Age category**				
	**20-34 (*n* = 862)**	**35-44 (*n* = 883)**	**45-54 (*n* = 690)**	**≥55 (*n* = 379)**	***P***
**Sex**					.20
**Women**	462 (53.60)	465 (52.66)	395 (57.25)	195 (51.45)	
**Men**	400 (46.40)	418 (47.34)	295 (42.75)	184 (48.55)	
**Education**					<.01
**High school or below**	167 (19.37)	217 (24.58)	263 (38.12)	229 (60.42)	
**College or above**	695 (80.63)	666 (75.42)	427 (61.88)	150 (39.58)	
**Occupation**					<.01
**Managers, administrators**	17 (1.97)	68 (7.70)	67 (9.71)	33 (8.71)	
**Professional**	155 (17.98)	141 (15.97)	103 (14.93)	50 (13.19)	
**Nonmanual, skilled**	210 (24.36)	203 (22.99)	148 (21.45)	73 (19.26)	
**Nonmanual, unskilled**	381 (44.20)	318 (36.01)	241 (34.93)	136 (35.88)	
**Manual, skilled**	42 (4.87)	45 (5.10)	37 (5.36)	25 (6.60)	
**Manual, unskilled**	57 (6.61)	108 (12.23)	94 (13.62)	62 (16.36)	
**Shift work**					<.01
**Yes**	181 (21.00)	129 (14.61)	101 (14.64)	56 (14.78)	
**No**	681 (79.00)	754 (85.39)	589 (85.36)	323 (85.22)	
**Psychological job demand**					<.01
**Low**	401 (46.52)	380 (43.04)	356 (51.59)	243 (64.12)	
**High**	461 (53.48)	503 (56.96)	334 (48.42)	136 (35.88)	
**Job control**					.01
**Low**	391 (45.36)	412 (46.66)	327 (47.39)	210 (55.41)	
**High**	471 (54.64)	471 (53.34)	363 (52.61)	169 (44.59)	
**Workplace violence**					.08
**Yes**	105 (12.18)	131 (14.84)	112 (16.23)	63 (16.62)	
**No**	757 (87.82)	752 (85.16)	578 (83.77)	316 (83.38)	
**Job insecurity**					.03
**Yes**	219 (25.41)	198 (22.42)	200 (28.99)	100 (26.39)	
**No**	643 (74.59)	685 (77.58)	490 (71.01)	279 (73.61)	
**Poor mental health**					.13
**Yes (BSRS-5 ≥6)**	110 (12.76)	124 (14.04)	86 (12.46)	35 (9.23)	
**No (BSRS-5 <6)**	752 (87.27)	759 (85.96)	604 (87.54)	344 (90.77)	
**Burnout** ^**b**^					.01
**Yes (≥50)**	104 (12.06)	135 (15.29)	108 (15.65)	35 (9.23)	
**No (<50)**	758 (87.94)	748 (84.71)	582 (84.35)	344 (90.77)	
**Techno-insecurity**					.08
**Yes**	150 (17.40)	179 (20.27)	156 (22.61)	80 (21.11)	
**No**	712 (82.60)	704 (79.73)	534 (77.39)	299 (78.89)	
**Techno-strain**					<.01
**Yes**	133 (15.43)	199 (22.54)	162 (23.48)	79 (20.84)	
**No**	729 (84.57)	684 (77.46)	528 (76.52)	399 (79.16)	

Abbreviation: BSRS-5, Brief Symptom Rating Scale 5.

a Values are *n* (%).

b Burnout was assessed using the Copenhagen Burnout Inventory, with a score of ≥50 indicating burnout.

Poor mental health and burnout were more prevalent among participants who reported techno-insecurity and techno-strain compared with those who did not (Table 2). Results of adjusted logistic regression models showed that techno-insecurity was associated with a 1.8-fold increased risk of poor mental health (OR = 1.78; 95% CI, 1.37-2.30; *P* < .01; Table 2) and a 1.9-fold increased risk of burnout (OR = 1.87; 95% CI, 1.45-2.41; *P* < .01. Techno-strain was associated with 2.2-fold increased risks of having poor mental health (OR = 2.19; 95% CI, 1.68-2.85; *P* < .01) and burnout (OR = 2.20; 95% CI, 1.70-2.83; *P* < .01). When both techno-strain and techno-insecurity were present, the risk of poor mental health was 3.24 times higher, and the risk of burnout was 3.64 times higher.

**Table 2:** Odds ratios (ORs) and 95% CIs of techno-insecurity and techno-strain for health in logistic regression analysis.^a^

	**Outcome: poor mental health**					
	**Poor mental health case**			**Logistic regression analysis**		
	***n***	**(%)**	***P***	**OR**	**(95% CI)**	***P***
No techno-insecurity	239	(10.63)	<.01	1 (Reference)		
Techno-insecurity	116	(20.53)		1.78	(1.37-2.30)	<.01
No techno-strain	226	(10.08)	<.01	1 (Reference)		
Techno-strain	129	(22.51)		2.19	(1.68-2.85)	<.01
**Combined effect**			<.01			
Insecurity (−) strain (−)	188	(9.49)		1 (Reference)		
Insecurity (+) strain (−)	38	(14.56)		1.52	(1.03-2.25)	.04
Insecurity (−) strain (+)	51	(18.96)		2.16	(1.51-3.10)	<.01
Insecurity (+) strain (+)	78	(25.66)		3.24	(2.36-4.44)	<.01
	**Outcome: burnout**					
	**Burnout case**			**Logistic regression analysis**		
	***n***	**(%)**	***P***	**OR**	**(95%)**	***P***
No techno-insecurity	251	(11.16)	<.01	1 (Reference)		
Techno-insecurity	131	(23.19)		1.87	(1.45-2.41)	<.01
No techno-strain	235	(10.49)	<.01	1 (Reference)		
Techno-strain	147	(25.65)		2.20	(1.70-2.83)	<.01
**Combined effect**			<.01			
Insecurity (−) strain (−)	193	(9.75)		1 (Reference)		
Insecurity (+) strain (−)	42	(16.09)		1.69	(1.16-2.46)	.01
Insecurity (−) strain (+)	58	(21.56)		2.42	(1.71-3.43)	<.01
Insecurity (+) strain (+)	89	(29.28)		3.64	(2.68-4.95)	<.01

a Models are adjusted for sex, age, education, occupation group, shift work, psychological job demand, job control, job insecurity, and workplace violence.

**Figure 2:**
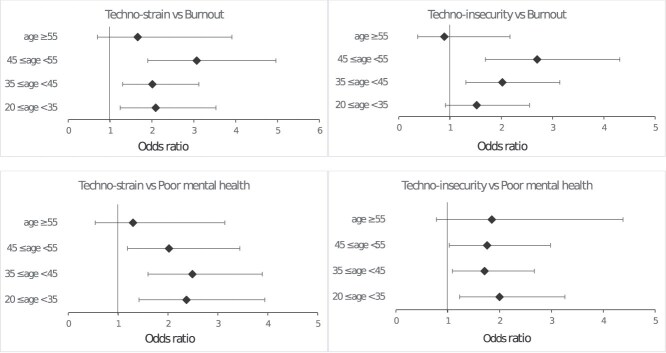
Odds ratios of techno-strain and techno-insecurity for burnout and poor mental health, stratified by age groups. Models are adjusted for sex, education, occupation categories, shift work, psychological job demand, job control, job insecurity, and workplace violence.

When study participants were stratified by age groups (Figure 2, Table S1), the association of techno-strain and techno-insecurity with burnout was prominent among middle-aged workers (45-54 years old). The associations of techno-strain and techno-insecurity with poor mental health were significant among workers younger than 55 years old. The associations were not significant among the oldest (≥55 years old) age group.

**Figure 3:**
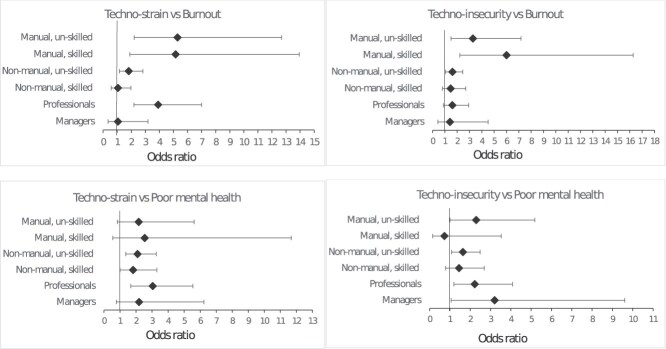
Odds ratios of techno-strain and techno-insecurity for burnout and poor mental health, stratified by occupational groups. Models are adjusted for sex, age, education, shift work, psychological job demand, job control, job insecurity, and workplace violence.

When study participants were stratified by occupational groups (Figure 3, Table S2), the association between techno-strain or techno-insecurity and burnout was significant among manual workers and low-skilled workers, whereas the association between techno-strain or techno-insecurity and poor mental health was significant among low-skilled workers and professionals.

## 4. Discussion

This study used a nationally representative sample of employees and discovered that techno-insecurity and techno-strain are associated with burnout and poor mental health. Techno-strain and techno-insecurity were associated with a 2-3-fold increased risk of burnout among middle-aged workers (35-55 years old), but no association was observed among older workers. Techno-strain and techno-insecurity were not associated with increased risk of poor mental health and burnout among workers older than 55 years. In regard to occupational groups, techno-strain and techno-insecurity were associated with burnout among manual workers, whereas techno-strain and techno-insecurity were associated with poor mental health among professionals and unskilled nonmanual workers.

The finding that both techno-strain and techno-insecurity were associated with higher risk of burnout and poor mental health is consistent with findings from studies regarding general job strain according to the JD-C model.^11^^,^^12^^,^^32^ Since we have included job control and psychological demands in the regression models, techno-strain and techno-insecurity represent additional work stressors due to technology in the modern workplace. Notably, technology is framed not only as a job demand but also as a job resource, such as autonomy and self-efficacy.^33^ In the current study, the measured techno-strain is presented as a negative subjective perception, and likely omits the consideration of technology as a resource at work. On the other hand, the mechanism of job insecurity is explained by the gain and loss spirals in Job Demands-Resources theory and Conservation of Resources theory. As individuals continue to lose resources, investment becomes more difficult.^34^ In the context of techno-insecurity, resources are being transferred from humans to new technology.

A novel finding of the current study is that middle-aged workers rather than older workers are at risk of burnout and poor mental health if exposed to techno-strain and techno-insecurity. An earlier study found that older workers experienced less technology-related strain compared with younger workers, such as feeling emotionally drained from technology use at work or fatigued by work assignments that involve the application of technology.^7^ However, the differential impact of technology-related strain on mental well-being across different age groups remains unclear. Notably, older workers who benefit from skill-upgrade training programs, where productivity is linked to technological advancements, tend to have a later intended retirement age.^35^ The healthy worker effect may explain the lack of associations of techno-strain and techno-insecurity with mental health risks in older workers in this study. In other words, senior workers who found it challenging to adapt to new technology may have already exited the workforce. Nevertheless, the workplace is an important setting to familiarize older adults with technology that may increase their digital literacy in later lives. Older adults who are skilled and fluent users of digital media became familiar with computers at an early stage of their working lives.^36^

Another novel finding is that occupational groups are differently influenced by techno-strain and techno-insecurity. Previous studies focused on small samples of managers observed a positive relationship of techno-insecurity with burnout.^37^ However, we observed a positive association between techno-insecurity and poor mental health among managers and professionals, but not between techno-insecurity and burnout. Managers’ and professionals’ work regularly involves rapidly evolving technology,^38^ which may lead to a mental health burden. However, in the current study the association of techno-strain and techno-insecurity with burnout was more prominent among manual workers, who are not familiarized with information and communication technology in their education and work career. It has also been observed that highly educated people adapt better to technology than less well-educated ones,^39^ suggesting that the divergence of educational content may lead to differences in using technology across occupational groups. Notably, in this study we measured poor mental health by the BSRS-5, which is designed to screen for depressive and anxiety disorders.^24^ Although there are overlaps in symptoms of burnout and mental disorders, the BSRS-5 measures individuals’ mood state in a general life context, whereas burnout measures exhaustion as a result of personal reaction to the work environment.^40^ The differential relationship across occupational groups could be due to a varied tendency to attribute and express one’s perception of techno-insecurity and techno-strain, as manual workers reported more fatigue symptoms than mood symptoms.

This study is strengthened by using a nationally representative sample across age and occupational groups, resulting in good generalizability. This study is one of only a few to focus on employees’ mental health using validated instruments. However, there are limitations to this study. First, the cross-sectional design of the survey prevents a causal inference between techno-stress and mental health. Reverse causation is possible, wherein workers with poor mental health may report higher levels of techno-insecurity and techno-strain. Second, we measured only partial aspects of techno-stress, omitting, for example, techno-invasion, techno-complexity, and techno-uncertainty. Furthermore, we used only 1 item to measure techno-insecurity and techno-strain; this has not been tested for its validity against the complete questionnaires. Therefore, caution should be observed when comparing findings from this study with other studies. In addition to techno-stress, technology has brought about positive influences in hedonic, eudemonic, and social/interpersonal domains.^5^ In this study, we did not consider the potential positive effects that technology may bring to the workplace, which could reduce work strain. Third, we did not assess individual factors that may moderate the association between techno-stress and mental health; for example, studies have shown that personality traits moderated the effect of techno-stress on burnout.^37^^,^^41^ This limitation restricts the study’s implications for personal intervention to promote workers’ mental health.

Techno-strain and techno-insecurity were associated with poor mental well-being among employees, but the association differed by age and occupation groups. Several studies have examined the role of employees’ personality for coping with technology^37^^,^^41^; nevertheless, techno-stress has been proposed as a manifestation of a lack of safety culture.^5^ Evidence shows that resources provided by the organization to help employees cope and work with technology moderate the effect of techno-stress on the well-being of employees.^42^ These resources may include training courses to help employees cope with new technology both in the skill aspect and the emotional aspect,^43^ improving workers’ self-efficacy. In addition to affecting workers’ health, techno-stress is linked to reduced productivity,^4^ as poor mental health is associated with productivity losses.^44^ In the near future, the impact of techno-stress as an occupational health issue is expected to increase. Although technology is designed to enhance productivity and efficiency, it is crucial for organizations to actively intervene to minimize techno-stress. Future studies, including intervention studies, are needed to explore how technology can be used to improve social integration and connectedness at work, enhance workflow to reduce workload, and promote positive emotions through an improved human–technology interface.^5^

## Supplementary Material

Web_Material_uiae079

## Data Availability

The data that support the findings of this study are available in the Opendata platform of the Ministry of Digital Affairs, Taiwan at https://data.gov.tw/en. These data were derived from the following resources available in the public domain: 111 year Occupational Safety and Health Survey (https://data.gov.tw/dataset/166056).
