# Supplementary material for: Techno-strain and techno-insecurity are associated with poor mental well-being in specific age and occupation groups
Source: J Occup Health. 2024 Dec 27;67(1):uiae079. doi: 10.1093/joccuh/uiae079 (PMC11827113; doi:10.1093/joccuh/uiae079)
Supplement: Web_Material_uiae079 [file web_material_uiae079.zip › Supplementary table.docx]

Supplementary table 1. Odds ratios of techno-strain and techno-insecurity for burnout and poor mental health, stratified by age groups. Models are adjusted for sex, education, occupation categories, shift work, psychological job demand, job control, job insecurity, and workplace violence.

|  | Predictor: techno-strain, outcome: burnout | | | |
| --- | --- | --- | --- | --- |
| Age group | OR | (95% confidence interval) | | p |
| 20 ≤ age < 35 | 2.09 | (1.24, | 3.53) | 0.005 |
| 35 ≤ age < 45 | 2.01 | (1.30, | 3.12) | 0.002 |
| 45 ≤ age <55 | 3.07 | (1.90, | 4.96) | <0.001 |
| age ≥ 55 | 1.66 | (0.70, | 3.91) | 0.250 |
|  | Predictor: techno-insecurity, outcome: burnout | | | |
| Age group | OR | (95% confidence interval) | | p |
| 20 ≤ age < 35 | 1.52 | (0.91, | 2.55) | 0.109 |
| 35 ≤ age < 45 | 2.02 | (1.31, | 3.14) | 0.002 |
| 45 ≤ age <55 | 2.70 | (1.69, | 4.31) | <0.001 |
| age ≥ 55 | 0.89 | (0.37, | 2.17) | 0.798 |
|  | Predictor: techno-strain, outcome: poor mental health | | | |
| Age group | OR | (95% confidence interval) | | p |
| 20 ≤ age < 35 | 2.37 | (1.42, | 3.94) | 0.001 |
| 35 ≤ age < 45 | 2.49 | (1.60, | 3.89) | <0.001 |
| 45 ≤ age <55 | 2.02 | (1.19, | 3.44) | 0.010 |
| age ≥ 55 | 1.30 | (0.54, | 3.14) | 0.554 |
|  | Predictor: techno-insecurity, outcome: poor mental health | | | |
| Age group | OR | (95% confidence interval) | | p |
| 20 ≤ age < 35 | 2.00 | (1.23, | 3.26) | 0.005 |
| 35 ≤ age < 45 | 1.71 | (1.09, | 2.67) | 0.019 |
| 45 ≤ age <55 | 1.76 | (1.03, | 2.98) | 0.037 |
| age ≥ 55 | 1.85 | (0.78, | 4.38) | 0.160 |

Supplementary table 2. Odds ratios of techno-strain and techno-insecurity for burnout and poor mental health, stratified by occupational groups. Models are adjusted for age, sex, education, shift work, psychological job demand, job control, job insecurity, and workplace violence.

|  | Predictor: techno-strain, outcome: burnout | | | |
| --- | --- | --- | --- | --- |
| Occupational group | OR | (95% confidence interval) | | p |
| Managers | 1.06 | (0.35, | 3.19) | 0.922 |
| Professionals | 3.91 | (2.19, | 7.00) | <0.001 |
| Skilled non-manual | 1.06 | (0.57, | 1.98) | 0.853 |
| Low-skilled non-manual | 1.82 | (1.17, | 2.83) | 0.008 |
| Skilled manual | 5.15 | (1.90, | 13.94) | 0.001 |
| Low-skilled manual | 5.29 | (2.21, | 12.68) | <0.001 |
|  | Predictor: techno-insecurity, outcome: burnout | | | |
| Occupational group | OR | (95% confidence interval) | | p |
| Managers | 1.41 | (0.44, | 4.51) | 0.559 |
| Professionals | 1.61 | (0.89, | 2.93) | 0.119 |
| Skilled non-manual | 1.47 | (0.80, | 2.70) | 0.212 |
| Low-skilled non-manual | 1.62 | (1.06, | 2.46) | 0.026 |
| Skilled manual | 6.00 | (2.22, | 16.27) | <0.001 |
| Low-skilled manual | 3.28 | (1.50, | 7.18) | 0.003 |
|  | Predictor: techno-strain, outcome: poor mental health | | | |
| Occupational group | OR | (95% confidence interval) | | p |
| Managers | 2.19 | (0.77, | 6.24) | 0.144 |
| Professionals | 3.04 | (1.67, | 5.54) | <0.001 |
| Skilled non-manual | 1.82 | (1.00, | 3.30) | 0.049 |
| Low-skilled non-manual | 2.10 | (1.36, | 3.26) | 0.001 |
| Skilled manual | 2.54 | (0.55, | 11.68) | 0.233 |
| Low-skilled manual | 2.17 | (0.84, | 5.62) | 0.111 |
|  | Predictor: techno-insecurity, outcome: poor mental health | | | |
| Occupational group | OR | (95% confidence interval) | | p |
| Managers | 3.20 | (1.06, | 9.61) | 0.039 |
| Professionals | 2.22 | (1.20, | 4.08) | 0.011 |
| Skilled non-manual | 1.45 | (0.78, | 2.69) | 0.245 |
| Low-skilled non-manual | 1.64 | (1.08, | 2.49) | 0.021 |
| Skilled manual | 0.73 | (0.15, | 3.53) | 0.700 |
| Low-skilled manual | 2.29 | (1.01, | 5.18) | 0.047 |
